# MIL-O-PD: a two-stage multiple instance learning and heuristic optimization framework for unpaired multimodal Parkinson's diagnosis

**DOI:** 10.3389/fnagi.2026.1924167

**Published:** 2026-09-09

**Authors:** Sankhadip Bera, Muhammad Fazal Ijaz, Jaeyoung Choi, Pawan Kumar Singh

**Affiliations:** 1 Department of Information Technology, Jadavpur University, Salt Lake Campus, Kolkata, West Bengal, India; 2 School of Technology, Business and Hospitality Faculty, Torrens University, Campus Flinders, Melbourne, VIC, Australia; 3 School of Computing, Gachon University, Seongnam-si, Republic of Korea

**Keywords:** attention mechanism, electroencephalography signals (EEG), gray wolf, multiple instance learning (MIL), Parkinson's disease

## Abstract

Parkinson's disease (PD) is a common neuro-degenerative disorder. Recent studies have used non-invasive biomarkers such as electroencephalography (EEG) and speech signals for PD diagnosis. However, majority of them optimize models at the instance level, whereas clinical diagnosis is a subject-level decision making process. In this work, we propose MIL-O-PD, a novel two-stage subject-level multimodal framework that formulates PD diagnosis. The stage-1 implements a Multiple Instance Learning approach with modality specific encoders and an attention-based aggregation mechanism. Stage-2 focuses incorporating a Gray Wolf Optimization-based *post-hoc* feature optimization module and final subject-level classifier. It supports representation-level multimodal fusion under strict subject-independent conditions. The proposed framework achieves up to 70.5% subject-level accuracy and 69.7% F1-score, while outperforming alternate pooling mechanisms. Further, the analysis of attention weights highlights modality-specific behavior, supporting the interpretability of the model. Overall, this study presents a principled framework for subject-level analysis of PD designed for unpaired multimodal data addressing the importance of aligning learning objectives with real-world diagnostic processes.

## 1 Introduction

**Figure 1:**
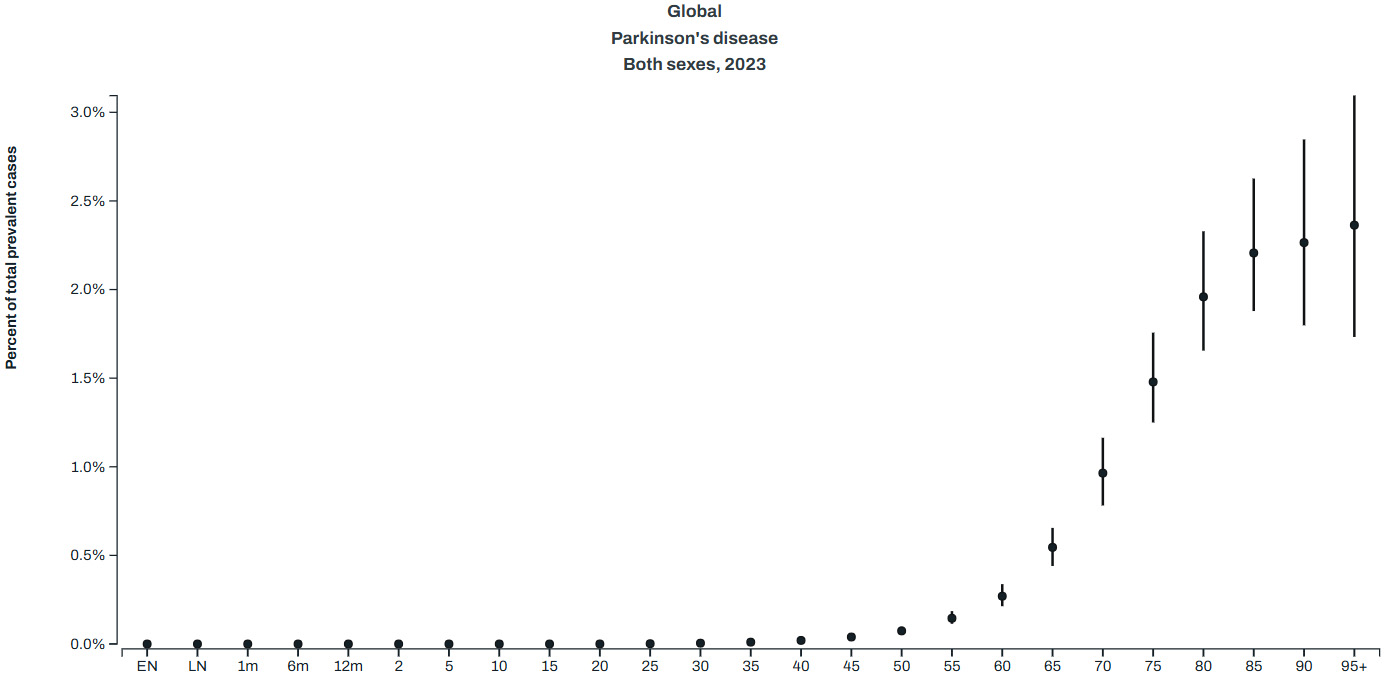
The prevalence of PD across the globe by age, for both male and female, in the year 2023 (vizHub, 2016). Source: Institute for Health Metrics and Evaluation (IHME), GBD Compare Data Visualization, University of Washington. Available online at: https://vizhub.healthdata.org/gbd-compare/.

Parkinson's disease (PD) is a neuro-degenerative movement-related disorder. It is primarily characterized by motor symptoms such as tremor, bradykinesia, rigidity, along with some non-motor indications including cognitive impairment, sleep disorders, depression, anxiety, etc. (Djaldetti et al., 2006; Moustafa et al., 2016). PD represents a growing public health challenge, with estimated prevalence ranging between 1% and 2% in persons over 60% and above 3% in those over 80 years (Sun et al., 2024). As per recent statistics, approximately 0.12% of the world population are living with PD (Luo et al., 2025). Figure 1 shows the prevalence of PD for different age groups in the year of 2023. The clinical diagnosis of PD still relies on patient-reported symptoms which can be subjective. The non motor symptoms often emerge in early and contribute substantially to disability and reduces quality of life. These symptoms are not systematically caught by routine physical assessments (Sun et al., 2024). So, there is an intense need of quantifiable biomarkers which can support earlier diagnosis and enable continuous monitoring of disease progression.

A wide range of physiological and digital modalities are explored as PD biomarkers, including neuro-imaging, wearable sensors (for gait and tremor), handwriting and drawing analysis, MRI scans and various biomedical and behavioral signals. Among these, electroencephalography (EEG) and speech or voice recordings are gaining interest because of their non-invasive nature (Anjum et al., 2024; Paul et al., 2025; Cao et al., 2025; Xu et al., 2025; Aich et al., 2025). They both are cost effective and can easily be accessible in both clinical and home environments (Bera et al., 2025; Saha and Singh, 2026; Sun et al., 2024; Bera and Singh, 2026). EEG can directly capture large-scale brain activities, while speech encodes complex interactions between motor control, phonation and high-level cognitive processes, making them complementary for PD like dysfunction.

Despite significant progress in PD analysis, a fundamental mismatch remains between how models are trained and how clinical decisions are made. Most approaches optimize performance at the segment level, whereas clinical diagnosis and decision making are subject-level processes. Although recent studies are adopting subject-independent evaluation protocols to handle data leakage, the training and inference are still predominantly being conducted at the segment level. However, not all segments contain disease related signatures, only few informative segments carry biomarkers relevant to PD. Many studies often follow heuristic aggregation schemes for decision making, are prone to error, as they may be inflated by misclassified noisy segments. Recent studies have increasingly adopted subject-independent evaluation protocols for EEG-based PD analysis. Protocols like leave-one-subject-out (LOSO), subject-level split or cross-validation on subject-level, address the concerns related to subject leakage between training and test splits and generate over-optimistic performance (Del Pup et al., 2025; Neves et al., 2024). With similar protocols and high-level feature extractions, Latifoğlu et al. (2024); Kurbatskaya et al. (2025) tried multiple machine learning models. Despite the subject-independent evaluation, most EEG-based approaches performed training and inference at the instance level. A small number of studies tries to aggregate epoch-level predictions through voting or fusion strategies. While Xiong et al. (2025), Mancazzo et al. (2026), and Wang et al. (2025) employed frame-level majority voting approach, Prieur-Coloma et al. (2026) used probability aggregation method to produce subject-level predictions for EEG-based PD diagnosis.

Speech-based studies of PD follow the same trend as EEG. However, truly subject-independent learnings are less common. Quan et al. (2022) maintained subject-independent splits on two speech datasets and implemented a combination of Convolutional Neural Networks (CNN) for binary PD classification. Ibarra et al. (2023) performed instance-level training using 1D,2D-CNN and Time-CNN-LSTM networks following the stratified subject-independent cross-validation method. However, the reliance on instance-level loss functions disconnects the learning objective from subject-level diagnosis. Speech-based subject-level studies for PD are less common. Siniukov et al. (2025) employed domain-adversarial learning for subject-level decision making via majority voting approach.

Multiple Instance learning (MIL) is an established approach and has wide application in biomedical domains where labels are naturally defined at a higher level than individual segments. In the thesis, Mahdizadeh (2024) investigated motor vigor in PD with MIL and an attention module using EEG signals. Though MIL is particularly appropriate for EEG and speech related studies, it has attracted very limited adoption in PD analysis (Jin et al., 2026; Xiao et al., 2025). Most existing PD studies that implicitly aggregate instances do not formulate the problem as MIL. Especially in multimodal framework, the subject-level learning remains weakly coupled to the training process.

Multimodal learning has been explored in PD research using combinations of speech, gait, handwriting or imaging modalities (Sar et al., 2025). However, joint modeling of EEG and speech under a subject-independent setting is largely unexplored. This absence highlights a major research gap.

These limitations motivate the need for a clinically realistic, multimodal and subject-level learning framework that is robust, stable and capable of making decisions directly at the subject-level.

### 1.1 Contributions

In this study, we introduce MIL-O-PD, a novel two-stage multimodal framework designed specifically for unpaired cohorts. While stage-1 focuses on subject-level representation learning within a Multiple Instance Learning (MIL) and attention pooling paradigm, stage-2 implements a post-hoc meta-heuristic feature optimization and final classifiers.

The specific contributions of our work are:

- We propose MIL-O-PD, a two subject-level, MIL-attention and Gray Wolf Optimization (GWO)-based framework specially optimized for unpaired multimodal data.
- We formulate EEG and speech analysis as bag-level learning problems, aligning training and inference to clinically realistic decision making.
- We provide a comprehensive stability analysis of stochastic meta-heuristic algorithms and comparisons with alternate selection methods.
- To the best of our knowledge, this is the first study to investigate PD employing a subject-level learning paradigm across both EEG and speech modalities.

**Figure 2:**
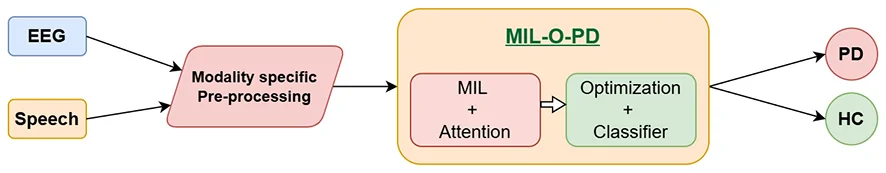
The high-level flow diagram of our proposed approach.

Figure 2 illustrates a high-level overview of our study.

## 2 Materials and methods

**Figure 3:**
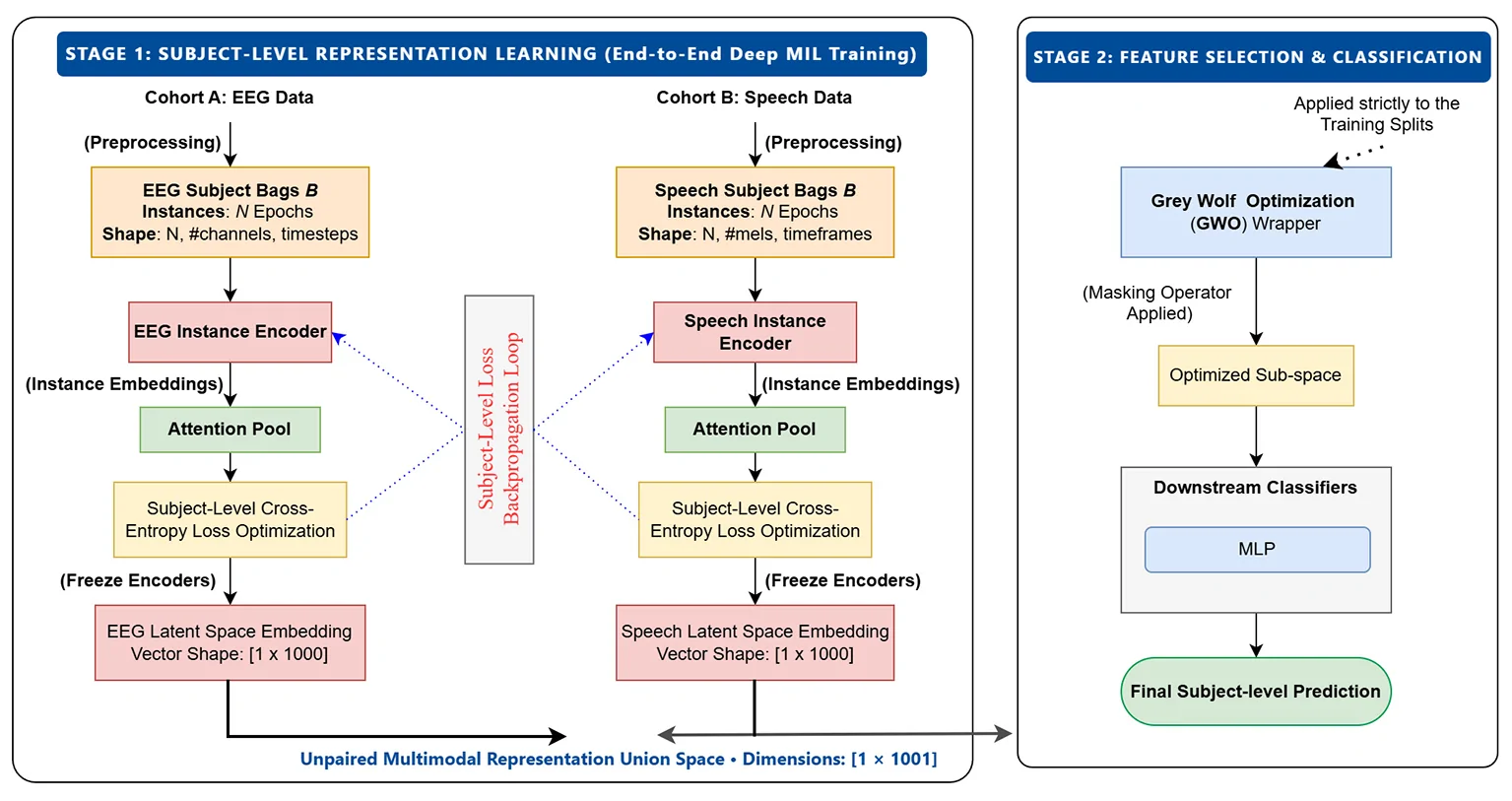
The overall architecture of MIL-O-PD framework.

In this section, we describe the proposed two-stage subject-level diagnostic framework, which aligns with the clinically realistic requirement. The following subsections describe details about the datasets, network configurations, experimental protocol used that comprise a complete end-to-end workflow. The overall architecture of our proposed MIL-O-PD framework is shown in Figure 3.

### 2.1 Datasets and pre-processing

In this study, we use two EEG and two speech datasets to evaluate our proposed framework. All datasets are publicly available on the internet. Table 1 summarizes the demography information for these datasets.

**Table 1:** Demographic summary of EEG and speech datasets used in this study.

	EEG		Speech	
	Dataset 1	Dataset 2	Dataset 3	Dataset 4
Subjects (PD/HC)	100/49	25/25	16/21	28/37
**Avg. age (PD/HC)**	68.5/70.9	69.7/69.3	–/–	67/(20,67)
**Gender (M/F)**	94/55	32/18	15/22	42/23

#### 2.1.1 EEG datasets

1. Dataset-1: The OpenNeuro-ds004584 dataset from OpenNeuro (Singh et al., 2023) includes resting state EEG recordings from 100 PD and 49 healthy controls (HC) subjects. All PD and HC subjects went through a single recording session. It has a total of 64 EEG channels and a sampling frequency of 500 Hz. Details about this dataset can be found in Anjum et al. (2024).
2. Dataset-2: The OpenNeuro-ds003490 dataset from OpenNeuro (Cavanagh, 2021) contains 64 channels EEG recordings from 25 PD and 25 HC subjects. The data were collected in the University of New Mexico in two sessions (with and without medications) for PD subjects and single session for HCs. Details about this dataset can be found in Del Pup et al. (2025).

We consider all subjects from dataset-1 and only with-medication session (all subjects) from dataset-2. The EEG pre-processing begins with retaining 29 common EEG channels from both the datasets. The channels included in this study are: “Fp1,” “AF3,” “F7,” “F3,” “FC1,” “FC5,” “T7,” “C3,” “CP1,” “CP5,” “P7,” “P3,” “O1,” “Oz,” “O2,” “P4,” “P8,” “CP6,” “CP2,” “C4,” “T8,” “FC6,” “FC2,” “F4,” “F8,” “AF4,” “Fp2,” “Fz,” “Cz.” All recordings are cropped to the first 120 s to focus on stable early segments, then a bandpass filtering between 0.5 Hz and 40 Hz is applied. The signals are then downsampled to 250 Hz, followed by average referencing of channels and an independent component analysis-based artifact removal process. Next, the cleaned data is segmented into 2-s segments with zero overlapping, yielding the shape of 29 (#channels), 500 (#timesteps) for each EEG segment. Then power spectral density (PSD) is extracted from EEG each segment using Welch's method, followed by log-transformation and per-channel *z*-score normalization (statistics computed only from training subjects and applied to validation and test data).

#### 2.1.2 Speech datasets

1. Dataset-3: The MDVR-KCL dataset (Jaeger et al., 2019), originated from King's College London Hospital in 2017, contains English voice recordings from 37 subjects, including 21 HCs and 16 PD patients. Subjects are asked to perform two different tasks: (a) read a pre-specified paragraph on a phone call, either “The North Wind and the Sun.” or “This is because there is less.” (b) One to one dialogue session with a doctor over the phone. Further information about this dataset can be found in Jaeger et al. (2019), Di Cesare et al. (2024).
2. Dataset-4: The IPVS dataset (Dimauro and Girardi, 2019; Dimauro et al., 2016) from IEEE contains voice recordings from 65 subjects in Italian language. The subjects are divided into three groups: 28 PD patients, 15 young HCs, and 22 elderly HCs. Recordings include some phonemically balanced text, phrases, words, and repeating syllables. Further information about this dataset can be found in Dimauro et al. (2016).

We use the text reading session for dataset-3 and dataset-4 (denoted as “B1” in the dataset, data available for 36 HCs and 16 PDs). Thus, the total number of subjects included under speech modality is 89 (32 PD/57 HC). Both datasets go through the same set of pre-processing pipelines. Audio recordings are first resampled to a common frequency value of 16 kHz. The amplitude peaks are normalized by dividing the signal by its maximum absolute value. To identify voiced regions and trim the silences, an energy-based voice activity detection (VAD) method is implemented using RMS energy computed with a frame length of 2,048 samples and hop length of 512 samples and a threshold of 0.01. The resulting signal is then segmented into 3-s segments with 1 second overlapping. Any remaining portions are then zero-padded to full length. Mel spectrograms (sample rate: 16 kHz, mel frequency bins: 128, and fast fourier transform with window size: 400 samples and hop length: 160 samples) are then computed from preprocessed segments. These representations (shape of: *#samples, #mels, #time*_*frames*) serve as inputs to the speech instance encoder. No other hand-crafted feature computation is performed.

### 2.2 Stage-1: Subject-level representation learning

Stage-1 is aimed to extract the subject-level embedding for both EEG and speech data. It implements a Multiple Instance Learning (MIL) framework with modality-specific instance encoders and an attention pooling mechanism to bag or subject-level representation learning.

#### 2.2.1 Multiple instance learning formulation

Clinical diagnosis with biomedical and speech signals operates at the subject-level, where the data are typically recorded as long continuous signals. But they are often segmented into multiple shorter segments for computational processing. Treating these segments as independently labeled samples may introduce a mismatch between the learning objective and the actual diagnostic task. MIL solves this issue by treating multiple segments as instances within a subject “bag” and a single label is associated with a bag full of instances (Dietterich et al., 1997; Fatima et al., 2023).

In MIL, each subject is represented as a bag:


$$\begin{array}{l} B_{i}=\left\{x_{i1},x_{i2},...,x_{iN_{i}}\right\} \end{array}$$

where *x*_*ij*_ denotes the *j*-th instance corresponding to EEG or speech signal of subject *i* and *N*_*i*_ is the number of instances available for that subject. A single label *y*_*i*_ is assigned to each subject or bag *B*_*i*_.

**Figure 4:**
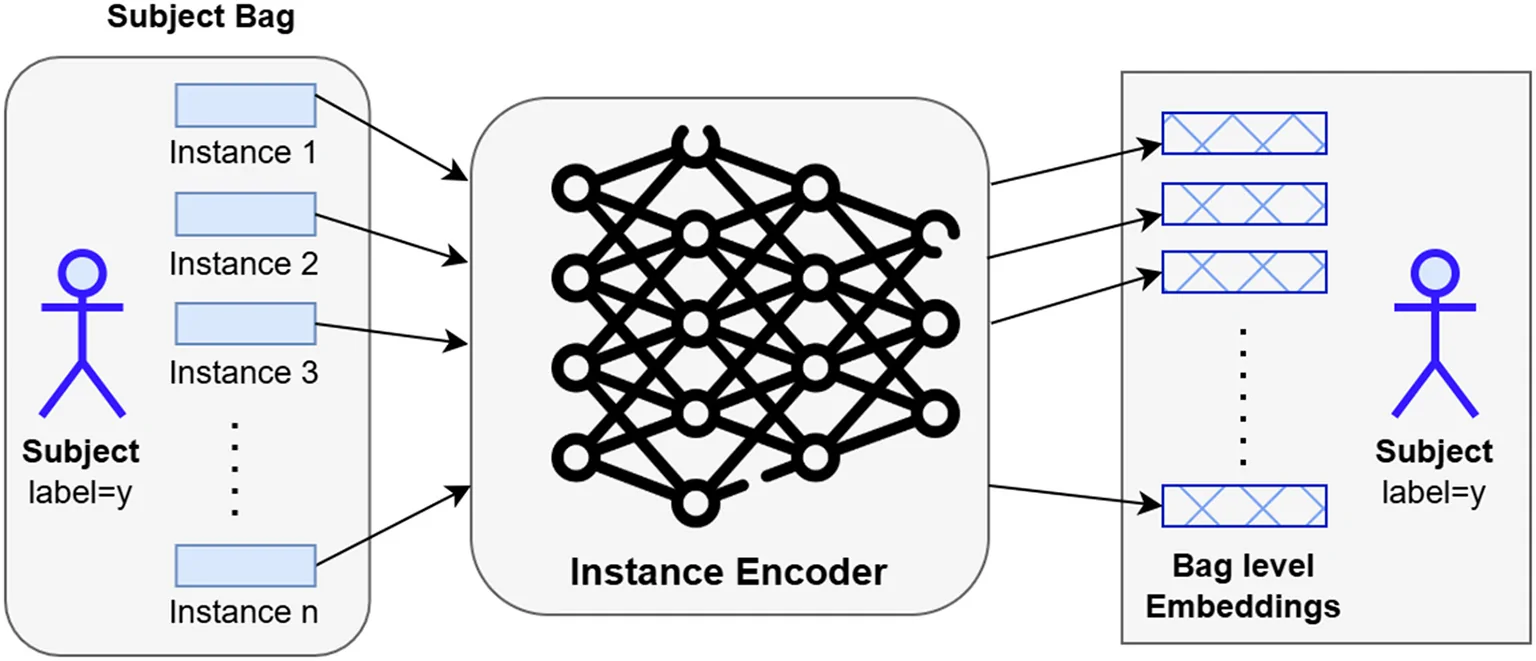
MIL for bag of instances.

A standard MIL framework is shown in Figure 4. It comes under weakly supervised learning approaches. Unlike traditional MIL assumptions that rely on the presence of at least one positive instance in the bag, the proposed framework adopts the representation-based formulation (Schmidt et al., 2024). All instances contribute to the subject-level decision with varying importance. The main goal here is to generate unique subject-level representation instead of instance-level representation.

#### 2.2.2 EEG instance encoder

Each PSD-based EEG instance has a shape of *C*×*F*, where *C* is the number of channels and *F* is the spectral dimension. The objective of the EEG instance encoder is to generate spatio-spectral representations from these EEG instances.

**Figure 5:**
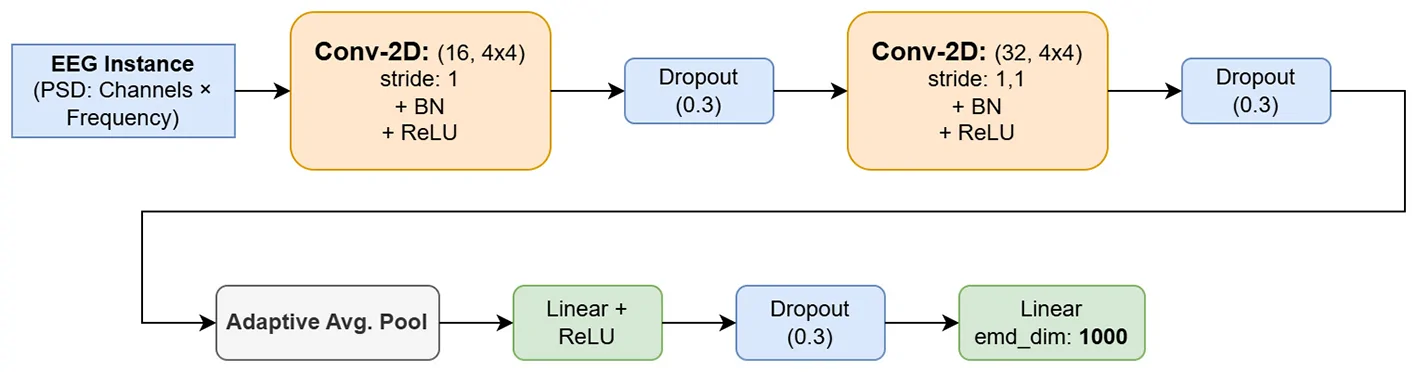
Architecture of the EEG instance encoder. The 2D convolutional network operates on channel-frequency representations, followed by global average pooling and two linear projection layers.

The EEG instance encoder consists of a sequence of blocks, each comprising a 2D convolutional and batch normalization layer followed by a ReLU activation and dropout. The convolutional kernels jointly operate across the channel and spectral dimensions, enabling the encoder to learn localized spatio-spectral patterns that are relevant for subject-level discrimination. After the convolutional stack, a global average pooling operation is applied. These pooled features are then projected through a fully connected layer to produce a fixed dimensional (1000) EEG embedding for each EEG instance. The architecture of the EEG instance encoder is shown in Figure 5. The encoder is shared across all subjects and instances, ensuring consistent representation learning under a complete subject-independent evaluation protocol.

#### 2.2.3 Speech instance encoder

**Figure 6:**
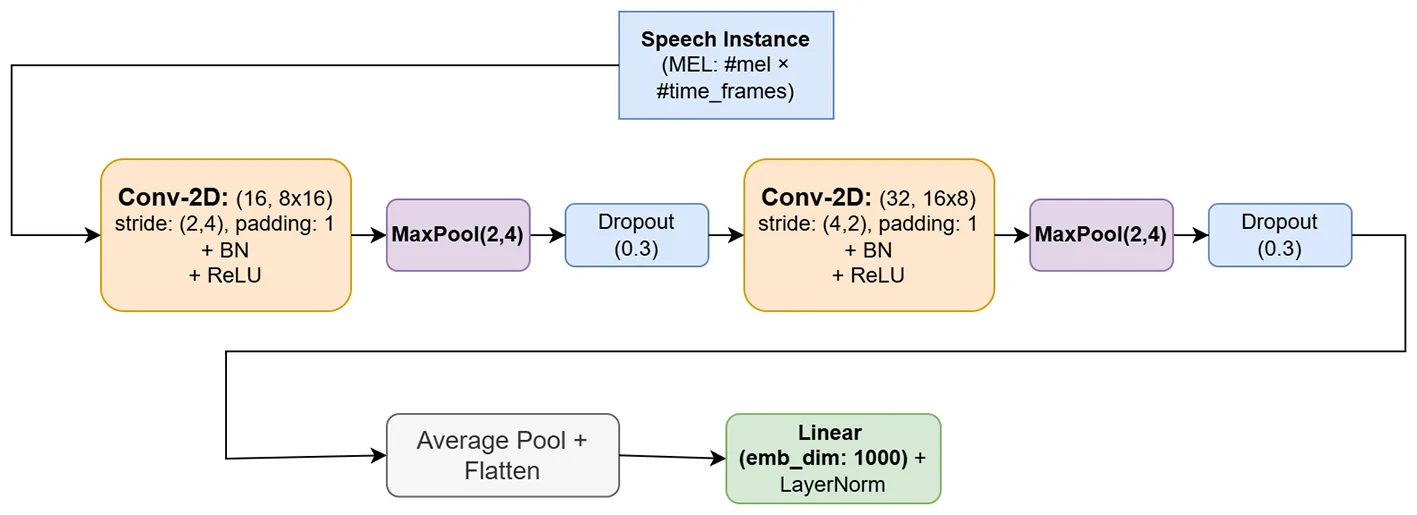
Architecture of the Speech instance encoder.

The speech instance encoder is also a standard CNN block. First, two 2D convolutional layers are used in conjunction with batch normalization and ReLU activations, followed by max-pooling and dropout to learn local time-frequency patterns from mel-spectrograms. After that, an adaptive average pooling and flattening operation is done. The final embedding has a fixed dimensionality (1,000) and serves as input to the attention-based instance aggregation module. Like the EEG encoder, the speech encoder parameters are shared across all segments and all subjects. The detailed architecture of the speech instance encoder is shown in Figure 6.

#### 2.2.4 Attention-based instance aggregation

A Bahdanau-style (Bahdanau et al., 2014) attention-based instance aggregation is used to perform soft instance selection, where the model assigns higher importance to informative instances and less weights to less relevant or noisy ones (Schmidt et al., 2024). Given a bag of instance embeddings {*e*_*i*1_, *e*_*i*2_, ..., *e*_*i*_*N*__*i*__} for subject *i*. An attention score is calculated for each instance using a trainable attention function:


$$\begin{array}{l} a_{ij}=v^{T}\tanh\left(W\cdot e_{ij}\right) \end{array}$$

where *W*∈ℝ^500 × *D*^ and *v*∈ℝ^500^ are trainable parameters. These scores are then normalized using a softmax function: $\alpha_{ij}=\frac{\exp\left(a_{ij}\right)}{\overset{N_{i}}{\underset{k=1}{\sum }}\exp\left(a_{ik}\right)}$, ensuring $\underset{j}{\sum }\alpha_{ij}=1$. The subject-level embedding *Z*_*i*_ for subject *i* becomes:


$$\begin{array}{l} Z_{i}=\overset{N_{i}}{\underset{j=1}{\sum }}\alpha_{ij}e_{ij} \end{array}$$

which is the weighted sum of the instance embeddings.

The attention mechanism is jointly trained with the respective instance encoders and the subject-level classifier in an end-to-end subject-level supervision. The loss function is defined at the subject-level (described in Section 4.5) and the attention weights are learned to optimize subject-level performance.

**Figure 7:**
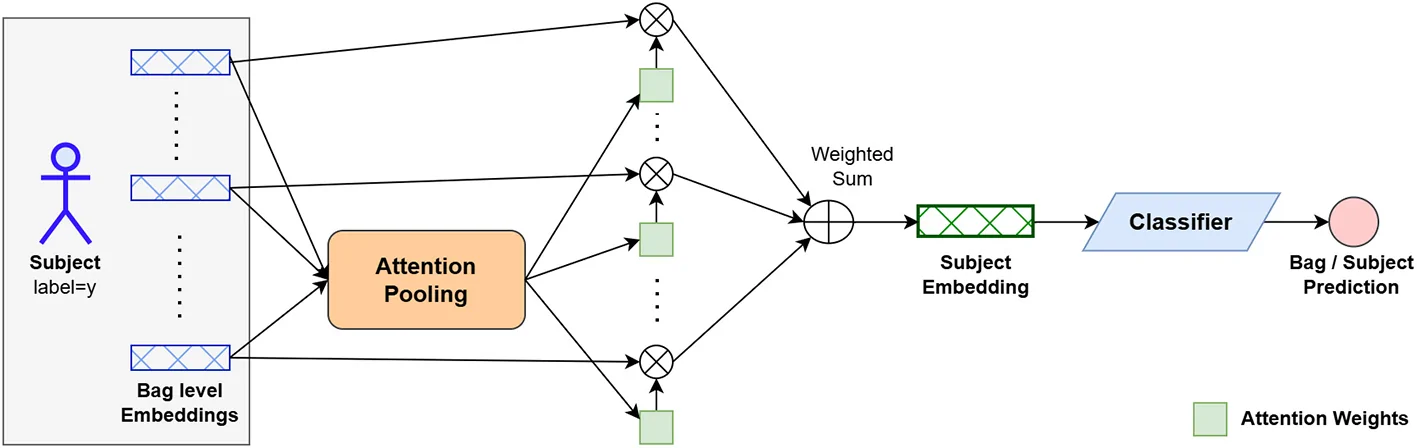
Architecture of attention pooling module.

Figure 7 represents the attention based instances aggregation used in our framework.

#### 2.2.5 Subject-level learning objective

A key aspect of the proposed methodology is to optimize the diagnostic performance at individual subject's level. After aggregating instance-level embeddings using attention pooling, an aggregated subject-level representation *Z*_*i*_ is obtained. The subject-level loss function using cross-entropy *loss* is defined as:


$$\begin{array}{l} loss=\frac{1}{N}\underset{i\in N}{\sum }CE\left(y_{i},\hat{y_{i}}\right) \end{array}$$

where *y*_*i*_ denotes the actual label for subject *i*, $\hat{y_{i}}$ is the predicted label, *N* is number of subjects in a batch and *CE* is cross-entropy.

This approach enables the attention mechanism to assign higher weights to informative instances and down-weight non-informative or noisy instances during training. Hence, the learning objective is minimizing the expected loss per subject. The overall training procedure for stage-1 is summarized in Algorithm 1

Algorithm 1 Subject-level MIL with attention pooling and cross-entropy loss (Stage-1). 
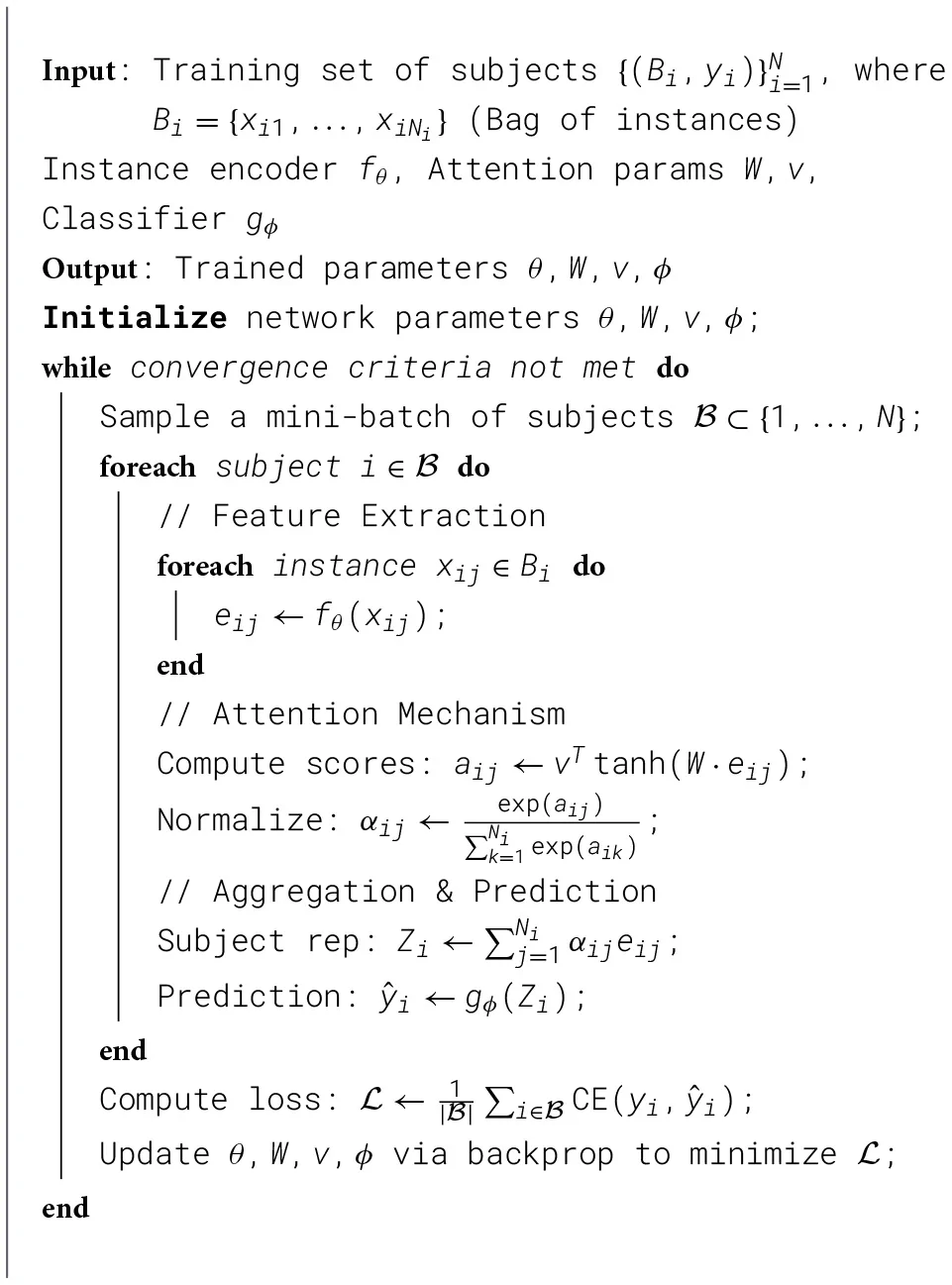

### 2.3 Stage-2: Representation fusion and meta-heuristic optimization

Stage-2 establishes a modular post-representation refinement workflow designed to leverage multimodal data. As both the modalities are structurally disjoint, the extracted subject-level embeddings via stage-1 are fused at the representation level. Then this joint latent space undergoes a post-hoc optimization process through meta-heuristic feature selection algorithm. Details about the fusion strategy and optimization methods are described below.

#### 2.3.1 Multimodal fusion

**Figure 8:**
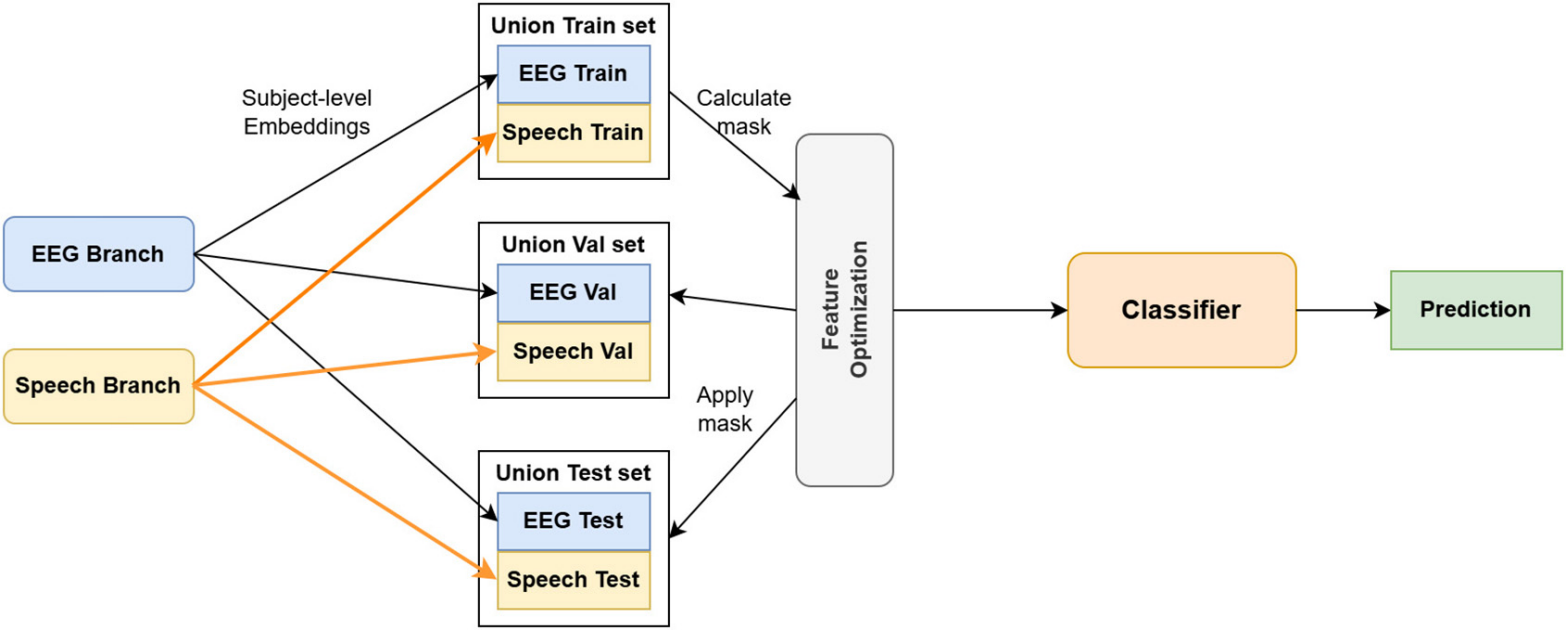
Fusion strategy.

After attention-based instance aggregation, each subject is represented by fixed-dimensional embeddings. These embeddings capture modality-specific diagnostic characteristics at the subject-level, forming the basis for multimodal fusion. As all the datasets are unpaired, that is, EEG and speech data come from different subjects, the multimodal learning is designed to operate at the joint representation level with a modality indicator (resulting 1,001 dimensional embedding per subject), allowing the classifier to learn modality-aware decisions. Importantly, the same train, validation and test splits from the respective modalities are maintained while creating this fusion set. The fusion strategy is shown in Figure 8.

#### 2.3.2 Feature optimization module

To minimize redundancies in high-dimensional subject-level embeddings, improve model generalization and enhance the discriminative quality of the final downstream classifier, we apply Gray Wolf Optimization (GWO) (Dhargupta et al., 2020), a meta-heuristic feature selection algorithm as a post-representation optimization module within the MIL-O-PD pipeline. GWO is a nature-inspired, population-based meta-heuristic optimization technique that mimics the social leadership hierarchy and hunting behavior of gray wolves (Hatta et al., 2019).

As these types of meta-heuristics algorithms are stochastic in nature, we use an adaptive consensus aggregation protocol. The optimization wrapper is executed for *N* = 5 independent stochastic runs on the same training features. For each latent dimension *i*∈{1, …, *D*}, a selection frequency score *f*_*i*_∈[0, 1] is computed as:


$$\begin{array}{l} f_{i}=\frac{1}{N}\overset{N}{\underset{m=1}{\sum }}M_{m,i} \end{array}$$

where *M*_*m, i*_∈{0, 1} be the binary selection status of the feature *i* in run *m*. We then formulate a data-driven, adaptive threshold (τ), dynamically computed by the mean pairwise Jaccard Stability Index (JSI, discussed later) across optimization runs: τ = 1−*J*. The final feature mask *M*^consensus^∈{0, 1}^*D*^ is defined as:


$$M_{i}^{\text{consensus}}=\{\begin{array}{ll} 1, & \text{if }f_{i}\geq \tau \\ 0, & \text{otherwise} \end{array}$$

This adaptive formulation directly couples feature selection to the search space stability. When *JSI* is high, τ becomes more permissive, preserving most of the latent features. Conversely, when *JSI* is lower, τ tightens adaptively and prunes unstable feature dimensions.

To ensure a fair evaluation, the GWO optimization is performed exclusively on the training set, and the selected mask is applied to the validation and test sets. Importantly, the optimization operates on the fixed subject-level embeddings generated by the end-to-end training of MIL framework. It acts as a representation refinement step without altering the core subject-level learning paradigm of MIL-O-PD.

### 2.4 Experimental design

All experiments are conducted under a subject-independent setting. For both modalities, the data are partitioned at subject-level, ensuring that no subject appears simultaneously in the training (60% subjects), validation (20% subjects), and test (20% subjects) sets. Subject-level embeddings from each modality are first learned independently using the proposed stage-1 algorithm. Table 2 summarizes the hyperparameter used during end-to-end training of instance encoders and the attention pooling layers.

**Table 2:** Hyperparameter specifications for Stage-1.

Hyperparameter	Value/setting
Optimizer	Adam
Learning rate	1 × 10^−4^
Weight decay	1 × 10^−4^
Epochs	50
Accelerator	T4 GPU (Kaggle)
Random seed	42

Since the EEG and speech datasets consist of disjoint subject populations, the multimodal evaluation is performed at the representation level. The extracted EEG and speech embeddings are combined into a union dataset with a modality indicator, preserving the original training, validation and test partitions within each modality. Further heuristic feature optimization is applied using Py-FS (Guha et al., 2021) framework to improve classification performance. These algorithms are applied independently on the training set and evaluated on the corresponding validation and test sets. Table 3 contains hyperparameter values used for the GWO optimization based on standard recommendations from the Py-FS framework (Guha et al., 2021). A multilayer perceptron (MLP) model is then trained and evaluated on the resulting sets for final classification.

**Table 3:** Hyperparameter specifications for Gray Wolf Optimizer (GWO) using Py-FS framework.

Parameter	Value	Description
Population size	20	Number of candidate solutions
Maximum iterations	50	Number of optimization iterations
Fitness function	Accuracy	Validation accuracy of classifier
Weight to accuracy	1	–
Number of runs	5	Number of Independent runs
Random seed	42	Ensures reproducibility

### 2.5 Evaluation metrics

Consistent with the clinical diagnosis, performance is evaluated exclusively at the subject-level. Standard classification metrics such as accuracy, macro F1-score and ROC-AUC are employed alongside 1,000-iteration non-parametric bootstrap 95% confidence intervals (CIs). The optimization stability across all independent stochastic runs is quantified using the Pairwise Jaccard Stability Index (*JSI*):


$$\begin{array}{l} J\left(S_{a},S_{b}\right)=\frac{\left|S_{a}\cap S_{b}\right|}{\left|S_{a}\cup S_{b}\right|}, \end{array}$$

where *S*_*a*_ and *S*_*b*_ are the selected feature subsets for run *a* and run *b*, respectively.

The average pairwise score across all 52 combinations is reported and utilized to calculate the adaptive threshold.

## 3 Results

This section presents a comprehensive empirical evaluation of our proposed two-stage framework across three distinct environments: EEG, speech alone, and their combined multimodal representation space. First, we present a detailed comparative analysis between unoptimized feature space and post-hoc GWO-based feature optimization masks along with stability analyses. Then a comparison with the standard selection method and an ablation study with alternative meta-heuristic search operators are discussed. Finally, the section concludes with the visual decoding of the underlying attention pooling mechanics used in stage-1.

### 3.1 Unimodal evaluation

Table 4 shows the performance comparison between full (1,000-dimensional) and optimized feature spaces across the individual modalities—EEG and speech.

**Table 4:** Unimodal performance on full and optimized (GWO) feature space with MLP.

	EEG only		Speech only	
	Full space	Optimized space	Full space	Optimized space
Accuracy [95% CI]	0.446[0.300, 0.600]	0.595[0.425, 0.750]	0.662[0.444, 0.889]	**0.718[0.500, 0.944]**
F1-score [95% CI]	0.438[0.275, 0.596]	**0.532[0.375, 0.702]**	0.644[0.411, 0.875]	**0.696[0.444, 0.935]**
ROC-AUC [95% CI]	0.385[0.208, 0.587]	**0.512[0.323, 0.715]**	0.734[0.389, 0.987]	**0.719[0.417, 0.974]**
Retained features (*K*)	1,000	**246**	1,000	**274**

ROC-AUC stands for Receiver Operating Characteristic-Area Under the Curve.

For EEG-only feature set, the unoptimized representations (*K* = 1, 000) evaluated via MLP yield moderate baseline performance (44.6% Accuracy, 43.8% F1-score, and 0.385 ROC-AUC). In Stage-2, GWO-based feature optimization is introduced to improve computational efficiency, eliminate redundant features, and enhance classification performance. With the compressed feature space (*K* = 246), the classification performance improves in all three metrics to 59.5% accuracy, 53.2% F1-score and 0.512 ROC-AUC. Similar trends can be seen for the speech-only cohort. On the full feature set (*K* = 1, 000), MLP achieves 66.2% accuracy, 66.4% F1-score and an ROC-AUC of 0.734. Stage-2 refined the representation to *K* = 274 features, boosting accuracy to 71.8% and F1-score to 69.60%, while a slight decrease (2%) in ROC-AUC is noticed.

Overall, while the unimodal evaluations demonstrate moderate predictive performance (maximum accuracy of 59.5% on EEG and 71.8% on speech), these conservative metrics directly reflect the strict subject-independent evaluation protocol and the inherent limitations of unimodal decision-making.

### 3.2 Results on multimodal fusion representation

As the EEG and speech datasets are unpaired, the multimodal integration is evaluated at the joint representation level (*D* = 1, 001, with a modality indicator). The comparative results on the multimodal fusion set before and after Stage-2 optimization are summarized in Table 5.

**Table 5:** Performance on multimodal union representation (Full vs. GWO).

	Optimization strategy	
	Full space	**GWO**
Accuracy [95% CI]	0.669[0.534, 0.793]	0.705[0.586, 0.810]
F1-score [95% CI]	0.640[0.504, 0.770]	0.697[0.575, 0.810]
ROC-AUC [95% CI]	0.637[0.482, 0.783]	0.740[0.606, 0.862]
Selected features (*K*)	1,001	729

Operating on the raw 1,001-dimensional union space, the classifier initially experiences a performance bottleneck, which can be due to the cross-modal structural noise, achieving 66.9% accuracy [95% CI: 0.534, 0.793], 64.0% F1-score [95% CI: 0.504, 0.770] and an ROC-AUC of 0.637 [95% CI: 0.482, 0.783]. In stage-2, the proposed GWO Consensus Mask (*K* = 729) successfully regularizes the latent space, improving performance across all evaluation metrics, elevating accuracy to 70.5% [95% CI: 0.586, 0.810], F1-score to 69.7% [95% CI: 0.575, 0.810], and ROC-AUC to 0.740 [95% CI: 0.606, 0.862]. The upward shift is also noticed in the lower bounds of the 95% CIs (5%–12%), confirming that the proposed feature selection significantly enhances model reliability under unpaired cross-modal evaluations. Although speech-only cohort achieves a little high accuracy (71.78%), it suffers from wide CIs ([0.500, 0.944]), while the multimodal union space provides a tighter and higher lower-bound confidence interval for all three metrics. Hence, integrating complementary modalities stabilizes the model against individual dataset shifts and avoids over-fitting to a specific recording environments.

#### 3.2.1 Analysis of optimization stability

We conduct an in-depth stability analysis of the GWO wrapper across five independent stochastic runs to evaluate its stochastic property. We measured the pairwise Jaccard Stability Index (JSI) to quantify feature subset consistency across all optimization runs. Only feature dimensions selected in ≥(1−*JSI*)% of independent optimization executions are retained in the final mask. Table 6 summarizes the stability metrics across the EEG, Speech, and Joint Fusion datasets.

**Table 6:** Feature optimization stability analysis for GWO across five independent runs.

Dataset cohort	Baseline dimension	Individual run *K* range [*Min, Max, Mean*]	Consensus *K*	Pairwise JSI
EEG only	1,000	306, 644, 549.4	246	0.3624
Speech only	1,000	406, 615, 565.6	274	0.3884
Fusion	1,001	599, 655, 629	729	0.4585

The pairwise JSI increases from unimodal sets (0.3624 on EEG, 0.3884 on Speech) to the joint fusion space (0.4585). This indicates that GWO converges significantly when complementary modalities are unified, as the model has access to diagnostic evidence from both modalities. While individual stochastic runs show variation in selected feature counts (e.g., *K*∈[599, 655] on Fusion), the consensus aggregation protocol effectively resolves the stochasticity, yielding a single, deterministic feature mask (*K* = 729) with superior generalization capability.

### 3.3 Comparative analysis with standard selection baselines

We compare the GWO-based selection against standard feature selection methods such as ANOVA *F*-Test, mutual information (MI), and Recursive Feature Elimination with Logistic Regression (RFE-LR). For fair comparison, all baselines are configured to select the same number of features (*K* = 729) as the GWO consensus mask. Table 7 summarizes the comparative benchmark. Both the statistical filters experience performance degradation compared to the unoptimized baseline (63.42% Accuracy with ANOVA F-Test and 63.64% accuracy with mutual information). The deterministic RFE-LR yields competitive performance (68.66% accuracy, 0.731 ROC-AUC). On the other hand, our proposed GWO-based optimization demonstrates the best overall performance (70.5% accuracy, 69.7% F1-score and 0.740 ROC-AUC), outperforming all other methods. The results prove that population-based global search is superior in regularizing non-linear latent spaces.

**Table 7:** Comparison with baseline feature selection methods with GWO on fusion space.

Method	Selection category	Retained features	Accuracy [95% CI]	F1-score [95% CI]	ROC-AUC [95% CI]
Full latent space	–	1001	0.669[0.534, 0.793]	0.640[0.504, 0.770]	0.637[0.482, 0.783]
ANOVA *F*-test	Filter	729	0.634[0.517, 0.758]	0.610[0.477, 0.739]	0.680[0.533, 0.811]
MI	Filter	729	0.636[0.500, 0.742]	0.605[0.464, 0.732]	0.616[0.447, 0.767]
RFE-LR	Wrapper	729	0.687[0.569, 0.810]	0.655[0.517, 0.776]	0.731[0.593, 0.852]
GWO consensus	Meta-heuristic	729	0.705[0.586, 0.810]	0.697[0.575, 0.810]	0.740[0.606, 0.862]

### 3.4 Meta-heuristic ablation

Along with GWO, we test two more prominent meta-heuristic optimization algorithms—Harmony Search (HS) (Geem et al., 2001), and Particle Swarm Optimization (PSO) (Ghosh et al., 2019). Table 8 compares the optimization behavior, selection stability (via pairwise JSI), and classification performance across these algorithms. The HS and PSO sho relatively lower JSI (0.2443 and 0.3309), resulting in severe information loss and dropping accuracy to 53.09% and 61.78%, respectively. With the highest JSI of 0.4585, the GWO outperforms other two with significant margins across all metrics and maintains a balanced feature footprint (*K* = 729). These results confirm that the GWO performs consistently better in this setting and based on this observation, HS is adopted as the primary feature optimization module in our proposed framework.

**Table 8:** Performance comparison of GWO with other meta-heuristic algorithms.

Method	Pairwise JSI	No. of features (Consensus)	Accuracy [95% CI]	F1-score [95% CI]	ROC-AUC [95% CI]
PSO	0.2443	87	0.531[0.397, 0.655]	0.523[0.390, 0.654]	0.593[0.437, 0.733]
HS	0.3309	177	0.618[0.483, 0.741]	0.612[0.482, 0.739]	0.705[0.565, 0.834]
GWO	0.4585	729	0.705[0.586, 0.810]	0.697[0.575, 0.810]	0.740[0.606, 0.862]

### 3.5 Analysis of stage-1 attention pooling

**Figure 9:**
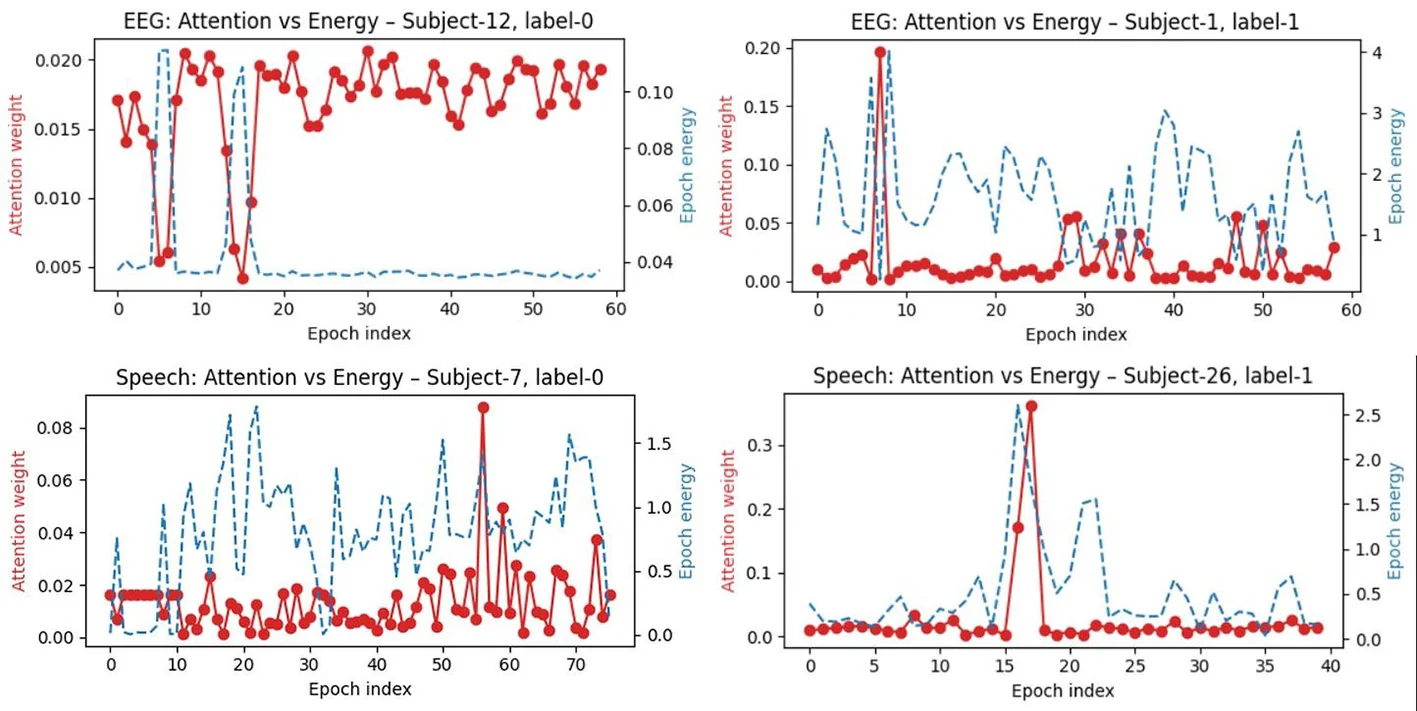
Attention weights vs. instance energy for EEG (upper row) and Speech (lower row).

To qualitatively validate the learned attention pooling mechanism, we compute the relationship between segment energy and attention weights for both EEG and speech modalities. In each subject, segments are sorted by attention weight and divided into a highly attended subset (top 10%) and the remaining. The average energy of these two groups is then compared. Figure 9 illustrates the relationships between attention weights and energy for both EEG and Speech modalities for both classes.

Interestingly, the attention mechanism adapts differently across modalities. For EEG, all subjects (100%) exhibit lower average signal energy in highly weighted segments compared to the remaining segments. The attention mechanism consistently suppresses high-energy segments, which may be associated with non-physiological muscle artifacts, eye blinks or movement-related artifacts, while focusing on quieter and structured activities (such as increased beta/alpha band power). But for speech, about 69% of subjects exhibit higher average signal energy in highly weighted segments. This finding is consistent with acoustic theory, where diagnostic information such as articulation, fundamental frequency perturbations, phonation, etc. are typically found in high-energy signals. These results strongly highlight the interpretability of the proposed framework, which is limited in regular approaches.

## 4 Discussion

The objective of our proposed work is to build a multimodal framework for PD diagnosis and to bridge the gap between instance-level learning paradigms and clinically realistic decision making. By formulating EEG and speech modalities as bag-level MIL problems and aggregating instances through attention pooling with an optimized subject-level loss, the model focuses on diagnostically informative segments while down-weighting noisy or less informative segments, providing a more clinically aligned learning objective than regular instance-wise training. The modality-specific encoders ensure that the learned representations capture both spatial (or frequency) and temporal dynamics that are relevant for PD pathology. To control the stochastic behavior of GWO, our Stage-2 framework introduces a data driven adaptive consensus protocol that directly relates feature retention to search space stability.

The experimental findings demonstrate that the proposed framework produces stable and meaningful subject-level representations across both modalities. Evaluated independently, unimodal representations exhibit poor performance (59.5% Accuracy on EEG and 71.8% on Speech) and relatively wider CIs, leaving unimodal classifiers vulnerable to site-specific noise. Fusion at the representation level further highlights the challenges of integrating heterogeneous and disjoint modalities. When these complementary latent representations are unified into a shared union space (*D* = 1, 001) and regularized via stage-2, the performance significantly improves. In the joint fusion space, the proposed stage-2 pipeline demonstrates a notable performance leap, driving subject-level accuracy to 70.5% [95% CI: 0.586, 0.810], F1-score to 69.7% [95% CI: 0.575, 0.810] and AUC to 74.0% [95% CI: 0.606, 0.862] (Table 5). The upward shift in the lower bounds of the bootstrap CIs validates that combining complementary physiological evidances reduces diagnostic uncertainty. The comparative analysis with standard selection methods and alternative meta-heuristic algorithms (HS and PSO) justifies the inclusion of the GWO-based feature optimization module in the proposed multimodal framework, highlighting its ability to produce stable and improved classification performance. Across multiple independent stochastic executions, the GWO achieved a superior pairwise JSI (*J* = 0.4585) compared to alternate meta-heuristics algorithms.

The analysis of attention pooling provides a notable insight into how the MIL mechanism adapts across modalities. For EEG, highly weighted segments exhibit lower average signal energy compared to the remaining segments in all subjects. This suggests that the model effectively learns to suppress high-energy EEG segments, which are often associated with artifacts or non-physiological noise, while prioritizing lower-energy but structured neural patterns. In contrast, the majority of subjects show higher energy in highly weighted segments, which aligns with the nature of speech processing, where high-energy segments usually contain the most information. This modality-dependent behavior supports that the attention weights are not random but consistent with known neuro-physiological and acoustic principles.

Overall, the findings demonstrate that subject-level MIL formulation along with multimodal fusion and optimizations provides a clinically aligned framework, offering improved stability and robustness.

### 4.1 Limitations

Despite its contributions, the present work has several limitations that should be acknowledged.

- The public datasets used in this work are limited in size, particularly for speech. While MIL is good for small samples, the limited subject diversity may restrict the capacity of deep encoders.
- The EEG cohorts have different acquisition sites and demography and the speech datasets span two different languages and distinct recording setups. Although subject-independent splits are enforced, without addressing these distributional shifts our framework may not generalize to other clinical settings.
- The multimodal fusion operates at the representation level due to the absence of paired EEG and speech data from the same subjects. As a result, the proposed fusion does not model cross-modal interactions.

## 5 Conclusion

In this work, we present MIL-O-PD, a principled two-stage framework for subject-level PD diagnosis, which generated up to 70.5% accuracy using EEG and speech signals. By formulating both modalities within a MIL paradigm, it explicitly addresses the misalignment between instance-level learning and subject-level clinical diagnosis while operating under strict subject-independent evaluation. The learned subject-level representations are stable and interpretable. Further, the attention analysis reveals modality-specific behavior consistent with known characteristics for both modalities. The representation-level fusion provides a practical solution for integrating heterogeneous and unpaired modalities, producing robust subject embeddings suitable for downstream analysis. Unlike traditional pooling methods, it provides better interpretability and remains free from the systematic biases generally encountered in those approaches. Overall, this study highlights the importance of aligning learning objectives with decision-making requirements for clinical situations and establishes it as an effective framework for subject-level neurological disorder analysis.

### 5.1 Future directions

For strengthening clinical relevance of our proposed framework, future directions should focus on:

- Evaluate the framework with larger and diverse cohorts, including more paired modality recordings.
- Exploring more explainability techniques, such as attribution-based methods, for better characterizing the neurophysiological and acoustic patterns associated with PD.
- Extending the binary classification to multi-class classification and disease severity estimation to enhance clinical relevance and applicability.
- Utilizing the learned subject-level embeddings for downstream clinical tasks such as biomarker discovery, personalized monitoring etc.

## Data Availability

Publicly available datasets were analyzed in this study. The EEG and Speech datasets used in this study are available in the following links: “OpenNeuro-ds004584: Available at OpenNeuro (v1.0.0)” OpenNeuro-ds003490: Available at OpenNeuro (v1.1.0) “MDVR-KCL: Available at Zenodo (Record 2867216)” IPVS: Available at IEEE Dataport.
